# Thermosensitive Injectable Hydrogels for Intra-Articular Delivery of Etanercept for the Treatment of Osteoarthritis

**DOI:** 10.3390/gels8080488

**Published:** 2022-08-05

**Authors:** Jomarien García-Couce, Timo Schomann, Chih Kit Chung, Ivo Que, Carla Jorquera-Cordero, Gastón Fuentes, Amisel Almirall, Alan Chan, Luis J. Cruz

**Affiliations:** 1 Biomaterials Center, University of Havana, Avenida Universidad Entre G y Ronda, Vedado, Plaza, La Habana 10400, Cuba; 2 Translational Nanobiomaterials and Imaging Group, Department of Radiology, Leiden University Medical Center, 2333 ZA Leiden, The Netherlands; 3 Percuros B.V., 2333 CL Leiden, The Netherlands

**Keywords:** osteoarthritis, etanercept, chitosan, Pluronic, thermosensitive hydrogels, intra-articular delivery

## Abstract

The intra-articular administration of drugs has attracted great interest in recent decades for the treatment of osteoarthritis. The use of modified drugs has also attracted interest in recent years because their intra-articular administration has demonstrated encouraging results. The objective of this work was to prepare injectable-thermosensitive hydrogels for the intra-articular administration of Etanercept (ETA), an inhibitor of tumor necrosis factor-α. Hydrogels were prepared from the physical mixture of chitosan and Pluronic F127 with β-glycerolphosphate (BGP). Adding β-glycerolphosphate to the system reduced the gelation time and also modified the morphology of the resulting material. In vitro studies were carried out to determine the cytocompatibility of the prepared hydrogels for the human chondrocyte line C28/I2. The in vitro release study showed that the incorporation of BGP into the system markedly modified the release of ETA. In the in vivo studies, it was verified that the hydrogels remained inside the implantation site in the joint until the end of the study. Furthermore, ETA was highly concentrated in the blood of the study mice 48 h after the loaded material was injected. Histological investigation of osteoarthritic knees showed that the material promotes cartilage recovery in osteoarthritic mice. The results demonstrate the potential of ETA-loaded injectable hydrogels for the localized treatment of joints.

## 1. Introduction

Osteoarthritis (OA) is the most prevalent chronic degenerative disease worldwide. It was estimated that globally 22.9% (corresponding to 654.1 million) of people over 40 years of age suffered from this disease in 2020 [1,2]. It is a disease that progressively affects the cartilage, synovial membrane, bone and periarticular tissues [2,3,4]. In joint diseases such as OA, the inflammatory process is the response of the immune system to injuries and when this is not resolved in time it becomes a chronic disease, causing intense pain and damage to the joints and surrounding tissues [3,5]. When the innate immune system recognizes the presence of damaged tissue, it activates the signalling pathways to produce proinflammatory effectors such as cytokines, chemokines, and prostanoids [5,6,7].

The management of OA involves pharmacological and non-pharmacological treatments which are applied mainly based on the seriousness of patients’ pain [4,8]. In general, in pharmacological treatments, the first option is to attack the symptomatic pain with the use of analgesics and non-steroidal or steroidal anti-inflammatory drugs [5,9]. In many cases, the efficacy is suboptimal. Its benefits are mainly limited by secondary effects such as gastrointestinal, renal and cardiovascular effects that occur as a consequence of the frequent administrations required [4,5]. Even if temporary relief of symptoms is achieved with these treatments, it is not possible to modify disease progression or prevent long-term disability. That is why, in recent years, many research works have been carried out on the development of disease-modifying drugs for OA (DMOADs). The goal of DMOADs is to modify the underlying OA pathophysiology and relieve associated structural damage to prevent long-term disability [10,11]. Several groups of molecules have been investigated, and among them, those that attack proinflammatory cytokines such as interleukin (IL)-1 and tumor necrosis factor-α (TNF-α) have shown promising advances [11]. TNF-α is one of the best characterized proinflammatory cytokines. It is known to stimulate the production of inflammatory mediators, and directly promotes the expression of matrix metalloproteinases (MMPs) and other matrix degrading enzymes involved in cartilage degeneration [11,12]. Etanercept (ETA) is a soluble fusion protein consisting of two 75 kD human TNFR IIs, each linked to an Fc portion of human IgG1 [13]. It was the first TNF inhibitor approved to treat rheumatoid arthritis in the United States in 1998 and in Europe in 2000 [1]. In more recent years, it was used to treat inflammatory OA. Ohtori et al. demonstrated in a 2015 study that a direct injection of ETA into OA knee joints could effectively relieve symptoms [14].

Intra-articular administration is an attractive alternative to traditional therapy for controlling OA symptoms. This is because by virtue of the localized nature of the disease, the drug can be administered directly at the main site where it has developed. However, its main disadvantage is that drugs are rapidly eliminated from the site of administration. This implies that multiple applications are required for an adequate therapeutic effect [3,7]. Consequently, repeated administration often leads to discontinuation of treatment by patients. As a solution to this, the incorporation of drugs into a sustained intra-articular release system has emerged as a strategy [15].

Due to their physical properties, hydrogels are excellent candidates for the development of intra-articular controlled release platforms. Their structural and mechanical similarity with the extracellular matrix gives them greater compatibility with cartilage tissue [16,17]. In situ formation hydrogels are those that can be administered as solutions or suspensions and the gelation process occurs due to the action of physicochemical factors such as pH or temperature. The so-called thermosensitive in situ hydrogels are those whose transition occurs due to the action of body temperature (37 °C) [15,18]. Poloxamers or pluronics are a family of triblock copolymers composed of hydrophilic (poly(ethylene oxide)-(PEO)) and hydrophobic (poly(propylene oxide)-(PPO)) segments distributed in chains in the form of PEO-PPO -PEO. The gel is formed from the self-assembly of the micelles formed by the PPO core and PEO crown under the action of temperature. Pluronic F-127 (PF) is most commonly studied for its application in controlled release systems due to its biocompatibility [19,20,21]; the main disadvantage of PF hydrogels is that they dissociate rapidly in aqueous medium, which can be solved by combining them with other polymers. Natural polymers such as chitosan, alginate, hyaluronic acid, and collagen, among others, have been used in conjunction with PF to prepare hydrogels [22]. Chitosan (CS) is a polysaccharide whose structure is highly similar to glycosaminoglycans that make up articular cartilage, so it has been frequently used in the development of hydrogels for intra-articular application. In addition, CS is a cationic, biocompatible, biodegradable polymer with marked mucoadhesive properties that are very useful for anchoring to surrounding tissue [2,23,24]. CS can also form gels by interacting with some salts such as phosphates, specifically with disodium β-glycerophosphate (BGP), to form thermosensitive hydrogels [25,26]. This system has also been extensively studied in the formation of injectable scaffolds for controlled release and tissue engineering.

Based on the above, injectable thermosensitive hydrogels were prepared from the physical mixture of PF and CS in this study, with BGP added for the intra-articular application of ETA. The aim was to verify that the hydrogels could achieve a controlled release of ETA and reduce damage to articular cartilage in the group of osteoarthritic mice treated with the system.

## 2. Results and Discussion

### 2.1. Hydrogel Preparation

**Figure 1:**
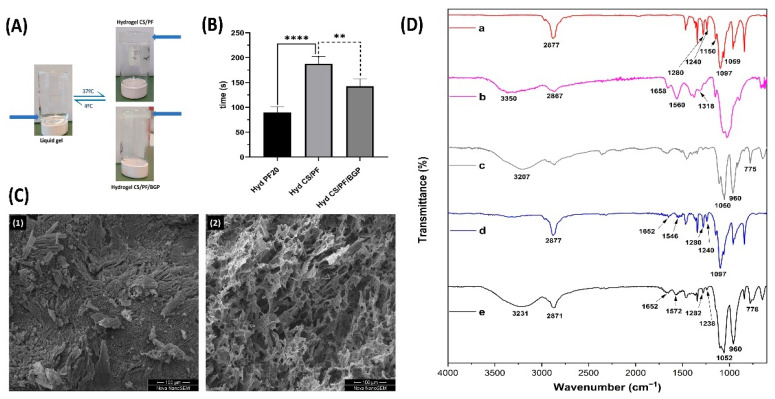
Characterization of CS/PF and CS/PF/BGP hydrogels. (**A**) Photographs of the CS/PF and CS/PF/BGP hydrogel transition from the liquid state at 4 °C (left) to the gel state at 37 °C (right). (**B**) Gelation times of CS/PF and CS/PF/BGP hydrogels at 37 °C. Values represent mean ± SD (*n* = 4, standard one-way ANOVA, ** *p* < 0.01 and **** *p* < 0.0001). (**C**) SEM of CS/PF (1) and CS/PF/BGP (2) hydrogels illustrating the microstructure. (**D**) FTIR spectra of PF (a), CS (b), BGP (c), CS/PF hydrogel (d), and CS/PF/BGP hydrogel (e).

Thermosensitive hydrogels were successfully prepared from the polymer mixture of CS and PF, while employing the chitosan solution as a base to dissolve the PF. The obtained solution was clear and transparent at low temperatures as observed in the bottle on the left of Figure 1A. The pH of this mixture was 6.2, a value that increased to 7.1 in the CS/PF/BGP sample due to the addition of BGP. The amount of BGP added to the polymeric solution did not introduce any visible changes before the gelation process. However, after increasing the temperature, the gel obtained was opaque with a whitish color in the CS/PF/BGP hydrogel, as can be seen in the lower bottle on the right in Figure 1A, compared to the upper bottle on the right which contains the hydrogel CS/PF which remains transparent.

#### 2.1.1. Gelation Time

The transition time from liquid to gel for thermosensitive, injectable hydrogels at the physiological temperature of 37 °C is a significant parameter for clinical applications. In practice, if the time is rapid, gelling will occur in the middle of the application process, which would result in clogging of the injection needle and prevent the material from reaching the desired location. On the other hand, if the gelation time is too long, diffusion of the occluded bioactive molecules or the gel itself will occur to areas outside of the intended destination. Therefore, the determination of the gelation time will assist in evaluating whether the designed hydrogel could be applied in practice.

As can be seen in Figure 1B, the CS/PF system has a longer gelation time compared to the 20% PF hydrogel sample used as a reference. The cause of this result is the hydrophobic interactions of the PF molecules, conditioned by the interspersed chitosan chains. This results in a slight decrease in the micellar formation and packing, which is the main cause of PF gelation [27,28,29]. In the CS/PF/BGP hydrogel, the mentioned effect was counteracted by the addition of BGP, which favored chitosan gelling when increasing the temperature to 37 °C. The gelation process that occurs with the increase in temperature after the addition of BGP to the CS solution is due to multiple simultaneous interactions between CS, BGP and water. The interactions that produce the sol/gel transition are as follows: (1) the increase in chitosan interchain hydrogen bonding as a consequence of the reduction in electrostatic repulsion due to the basic action of the salt, (2) the CS-BGP electrostatic attractions via the ammonium and the phosphate groups, respectively, and (3) the CS–CS hydrophobic interactions which should be enhanced by the structuring action of glycerol on water [30,31,32]. For those reasons, we concluded that there are two synergistic effects in the gelation process with the increase in temperature when BPG is added. These effects are the formation of PF micellar packing and the increase in hydrophobic and electrostatic interactions, as well as the hydrogen bonds between the CS chains produced by BPG [30,33,34].

#### 2.1.2. Morphological and Chemical Characterizations

Images taken from the freeze-dried hydrogels (Figure 1C) revealed that the surface morphology in the CS/PF samples (Figure 1C (1)) was compact with the presence of small channels. In contrast, in the CS/PF/BGP hydrogels (Figure 1C (2)) an interconnected porous morphology was observed, suggesting that the addition of the gelling agent created micro- and nano-scale pores in the structure [35]. As previously shown, the gelation time reduced with the addition of BGP because the hydrogen bridge bonds between the CS chains increase and electrostatic interactions arise between BGP phosphate groups and CS amino groups [30,36]. These same conditions cause the interaction between the polymer chains, which favors the development of pores in the structure. These pores are clearly visible in the micrograph (Figure 1C (2)). The presence of pores favors cell adhesion, absorption, and diffusion of nutrients, as well as the transport of waste through the matrix [37], which further improve the properties of the material to integrate into the implantation site.

FTIR spectroscopy was used to assess the functional groups characteristic of CS, PF, and BGP, and to verify the possible interactions between them. Figure 1D shows the ATR-FTIR spectra of the prepared hydrogels together with the starting materials. The FTIR spectrum of PF was characterized by several main absorption bands. The C-H stretch vibrations appeared at 2877 cm^−1^, the CH_2_ twisting vibrations that gave rise to two bands at 1240 and 1280 cm^−1^, and a typical triplet of overlapped intense bands (1059, 1097 and 1150 cm^–1^) characteristic for the C-O-C stretching signals were observed [38,39]. In the pure CS spectrum, a broadband between 3500 and 3200 cm^−1^ corresponding to the stretching vibrations of O-H and N-H and the absorption band in the region of 2940 to 2700 cm^−1^ with a maximum of 2867 cm^−1^ representing the symmetrical stretching of C-H in the pyranosic ring were distinguished [40,41]. Bands were also observed at 1658 and 1560 cm^−1^ corresponding to the C=O stretch of the amide bond (amide I) and the N-H flexion of the primary amine groups (amide II), respectively [41,42]. The peak at 1320 cm^−1^ was attributed to the C-N stretching in the secondary amide (amide III) [43]. In the BGP spectrum, two intense signals were detected, with one at 1050 cm^−1^ related to the asymmetric stretching vibrations of the PO_4_^3−^ group (ν_3_) and the other at 960 cm^−1^ related to the symmetrical stretching vibrations (ν_1_) of the same group [35,42,44]. There was another band of lower intensity due to the aliphatic stretching of P-O-C [32].

In the spectrum of the CS/PF hydrogel, signals associated with PF were mainly observed (Figure 1D), which was the main component in this formulation. In addition, many of the PF bands overlapped with those of the CS. However, some of the CS signals were visible such as amide I and amide II bands. However, it should be noted that they had undergone slight frequency changes at lower wavenumbers. The amide I band changed from 1658 to 1652 cm^−1^ and the amide II band from 1560 to 1546 cm^−1^, which was associated with hydrogen bond interactions between PF hydroxyl groups and CS amino groups. In the CS/PF/BGP hydrogel, the spectral characteristics differed slightly from the CS/PF hydrogel as expected due to the overlap considering the phosphates group of BGP. New bands appeared at 778 cm^−1^ and others underwent changes in position due to the interactions between chitosan and BGP. Unlike our observation in the CS/PF hydrogel, we detected a shift towards higher frequencies of the amide II band from 1560 to 1572 cm^−1^. This indicates there were interactions between the amino group of the CS and the phosphate group of the BGP due to the electrostatic forces or hydrogen bonds [35,41]. Further evidence of the formation of intermolecular interaction between CS and BGP during the process of CS/PF/GP hydrogel formation was obtained effectively by conducting an analysis of the infrared spectrum of the stretching vibrations of -OH groups and -NH_2_ groups. In our case, the OH absorption band was shifted to lower wavenumbers with a broad and blunt peak (from 3350 to 3231 cm^−1^), suggesting that some hydrogen bonds were formed in agreement with the reported work by Wang and Chen in 2015 [31].

### 2.2. In Vitro Experiments

#### 2.2.1. ETA Release Study

**Figure 2:**
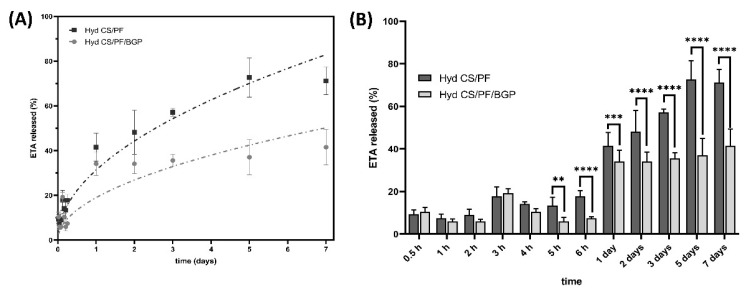
In vitro ETA release profile. (**A**) Release behavior of the ETA from CS/PF and CS/PF/BGP hydrogels. The error bars represent mean ± SD (*n* = 3). (**B**) Comparison of the amount of ETA released at each time point. Values represent mean ± SD (*n* = 3; two-way ANOVA, Sidak’s multiple comparisons test, ** *p* < 0.01, *** *p* < 0.001 and **** *p* < 0.0001).

To determine the ETA released from the CS/PF and CS/PF/BGP hydrogels in vitro, the cumulative release was measured at several time points. Values were calculated from the ratio between the mass of drug released and the total initial mass in the hydrogel multiplied by 100 to obtain percentage value. Figure 2A shows the cumulative release profiles of ETA at 37 °C in PBS (pH = 7.4) over 7 days. Additionally, the graph in Figure 2B depicts the obtained data in form of bars, which allows for a more detailed statistical analysis of the amount of ETA released over time. A sustained release over time could be observed in the profiles of both hydrogels. However, the overall release of ETA from BGP-containing hydrogels was sustained and comparatively slower than that of CS/PF hydrogels. After 7 days, CS/PF/BGP hydrogels released only 40% of ETA, while CS/PF hydrogels released 70% of ETA over the same duration. In the initial times, the release of ETA was similar in both hydrogels. This behavior can be explained by the fact that the ETA molecules closest to the surface are not limited by the entanglement of chitosan due to the action of BGP. Thus, ETA release depends only on the proportion of the main components of the system, which are similar. However, at later time points (3–7 days), a significantly reduced ETA release could be observed for CS/PF/BGP hydrogels.

According to the previous literature, it can be summarized that the morphology and size of the drug molecule, as well as the type and strength of its interactions with the matrix, are the main factors that affect its release from hydrogels [45,46,47,48]. Analyzing the first mentioned factor and considering that the morphological analysis showed that the incorporation of BGP generates a highly porous structure unlike that of the CS/PF hydrogel, we expected an increased ETA release from the CS/PF/BGP hydrogel. However, our result shows the opposite. It is noteworthy that ETA is a compound of high molecular weight (150,000 Da), which implies that the molecules that are trapped within the most tangled areas of the chitosan chains after the formation of the gel cannot easily migrate to the outside, thereby delaying the release process. Taking these elements into consideration, it can be deduced that the predominant factor in the release is not the porosity of the material, but rather the size of the occluded molecule.

Since cumulative drug release is a complex process, it can be the result of a conjunction of several phenomena, e.g., diffusion and swelling, among others. We studied four previously described mathematical models to investigate the possible mechanisms that could influence the release of ETA from our hydrogels. The values obtained from these mathematical models for the CS/PF and CS/PF/BGP hydrogels are listed in Table 1. First, the Higuchi equation was evaluated, which allowed us to quickly and easily estimate whether release occurs mostly through a Fickian diffusion process or not [49,50,51]. Subsequently, the Kormeyer–Peppas and Peppas–Sahlin models were applied to evaluate the diffusion of the drug from the matrix based on the incorporation of the diffusion exponent (*n*) into the calculation. The *n*-value indicates whether the release occurs by a process of diffusion or chains relaxation, or both simultaneously [49,52,53]. In addition, with the help of the Peppas–Sahlin model, it was possible to assess the predominant mechanism in the release process based on the constants *K*_1_ and *K*_2_.

As can be seen in the results shown in Table 1, the correlation coefficient (*R*^2^) has values greater than 0.95 in the CS/PF hydrogel for all the models studied, while in the case of the CS/PF/BGP hydrogel the values are lower than 0.90. This implies that in the first case, the fitting is good and the mechanisms described by the models are the ones that govern the release process. Therefore, the mechanism or mechanisms that influence the release process in the second case cannot be explained by the models studied under the developed working conditions due to the associated complexity.

However, a separate assessment must be made of each model, because each one has its peculiarities. For the CS/PF hydrogel, when making a global analysis considering the information provided by each model, it can be established that ETA release occurs by a diffusion process that we were unable to catalog as totally Fickian. The Higuchi model describes a purely Fickian diffusion and although the data obtained show a fairly good fit, it is not the model that has the *R*^2^ value closest to 1. On the other hand, in the Kormeyer–Peppas and Peppas–Sahlin models the values of *n* obtained were 0.41 and 0.56, respectively. The first value reports a quasi-Fickian diffusion. This implies that the process is also influenced by the nature of the hydrogel and the second indicates an anomalous type of transport where the release mechanism is a combination of diffusion and swelling. In the case of the CS/PF/BGP hydrogel, as seen before, the fitting was not good, but a comparison could be made using the Peppas–Sahlin model as a reference, which showed the best results.

When the results of this model were analyzed with more accuracy, it was found that the values of *K*_2_ were negative in both cases. This implies that the term of the equation does not have a physical meaning and the relaxation of the chains does not contribute to the release process. On the other hand, the value of *n* in both cases is very similar and greater than 0.5, as can be seen in the results shown in Table 1. This implies that the release is non-Fickian or anomalous and the drug is released by diffusion and swelling. Regarding the value of *K*_1_, which provides information on the kinetics of Fickian diffusion, it is seen that it is higher in the case of the CS/PF hydrogel, which implies that diffusion has a greater effect than in the case of the CS/PF/BGP hydrogel. These results are mainly conditioned by the same factor analyzed previously, i.e., the relationship between the molecular weight of ETA and the tangling of the chains that occurs when BGP is added to the system.

#### 2.2.2. Cell Viability and Proliferation

**Figure 3:**
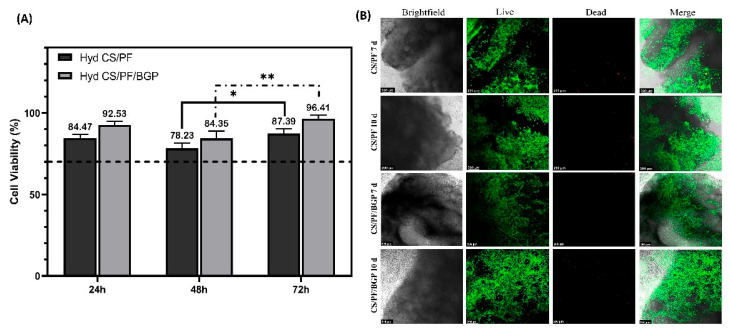
Viability assay and morphology of cells grown on hydrogels. (**A**) Cell viability of C28/I2 cells grown on CS/PF and CS/PF/BGP hydrogels. Values represent mean ± SD (*n* = 3; two-way ANOVA, Tukey’s multiple comparisons test, * *p* < 0.05 and ** *p* < 0.01). (**B**) LIVE/DEAD^®^ staining of chondrocytes seeded on CS/PF and CS/PF/BGP hydrogels after 7 and 10 days in culture. Scale bar = 200 µm.

An MTS assay was used to measure the cell viability and metabolic activity of chondrocytes cultured on the hydrogels for 72 h. The results (Figure 3A) show that the cell viability was above 78% after 24, 48, and 72 h for both samples when compared to C28/I2 cells in standard cell culture. Thus, according to the ISO 10993-5 [54], the results show that hydrogels are cytocompatible. In addition, the incorporation of BGP did not affect the viability of chondrocytes cultured on the hydrogel. Statistical analysis revealed a statistically significant increase in the cell viability between 48 and 72 h for both hydrogels, which indicated cell proliferation and reaffirmed the cytocompatibility of our hydrogels. A trend was visible when comparing CS/PF hydrogels with CS/PF/BGP hydrogels in that the viability of C28/I2 cells cultured in CS/PF/BGP hydrogels was constantly higher than that of cells cultured on CS/PF hydrogels. This might indicate BGP has a beneficial effect on cell viability. However, this trend was not statistically significant.

LIVE/DEAD staining was performed to investigate whether the hydrogels had no significant cytotoxic effects on the chondrocytes after 72 h of culture (Figure 3B). It can be seen that there was no noticeable presence of dead cells (red) and a high number of living cells (green) were distributed homogeneously over both hydrogels. In addition, the cell population increased between day 7 and day 10 of incubation, indicating that hydrogels permitted cell proliferation in vitro. In general, the results indicate that both hydrogels facilitate the adhesion, proliferation, and growth of C28/I2 cells over time. Furthermore, BGP did not adversely affect cytocompatibility.

### 2.3. In Vivo ETA Release Study

**Figure 4:**
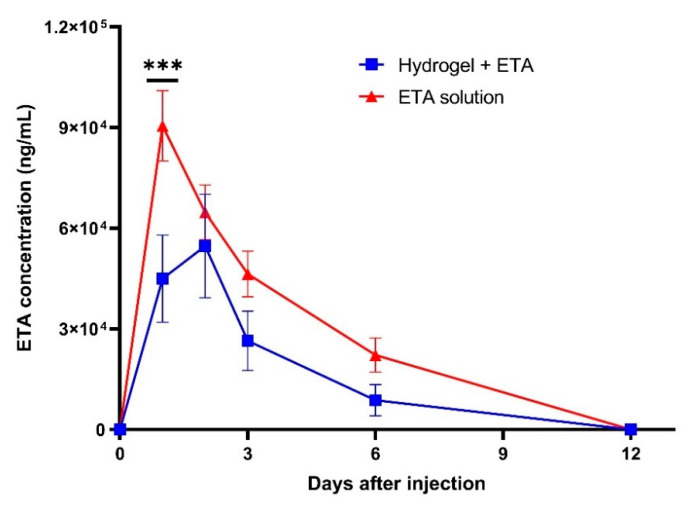
In vivo ETA release profiles. The red and blue curves represent the ETA solution and the hydrogel + ETA, respectively, injected in the knee joint of mice with OA. The error bars represent mean ± SD (*n* = 6). Statistical significance was determined by two-way ANOVA with multiple comparisons; *** *p* < 0.001.

The ETA release from intra-articularly injected CS/PF/BGP hydrogels was studied and compared to mice injected with ETA (100 µg/mL) in PBS. Blood samples were collected on day 1, 2, 3, 6, 9, and 12 after intra-articular injection and ETA concentrations were determined by means of ELISA. Figure 4 shows the ETA concentration in the plasma of the treated mice over time.

The maximum concentration value *(C_max_)* of ETA in mice injected with ETA in PBS was reached within the first 24 h, after which the concentration of ETA gradually decreased until day 12 showing a behavior similar to conventional drug delivery systems. The ETA release curve of hydrogel-injected mice was relatively similar to that of ETA in PBS.

However, the release from hydrogels had the following two distinct differences: (i) The amount of ETA released was lower, which might be due to the fact that a part of the ETA was retained within the hydrogel matrix. (ii) The *C_max_* of ETA was reached 48 h after the injection, indicating a sustained release of ETA. However, ideally, the drug release from the hydrogels should be more sustained resulting in an elevated concentration of ETA in the blood over time, as observed in our in vitro study. This indicates that a degradation/elimination process occurred in vivo. To overcome this condition, the administered dose needs to be sufficient to maintain a high drug concentration and release. Since a total of only 6 µL of ETA-loaded hydrogel was injected into the knee joints of animals, the administered ETA dose in this study was very small (0.6 µg). However, it was shown that the hydrogel regulates ETA release in vivo, indicating its potential as a controlled release system.

### 2.4. Near-Infrared (NIR) Fluorescent Imaging

**Figure 5:**
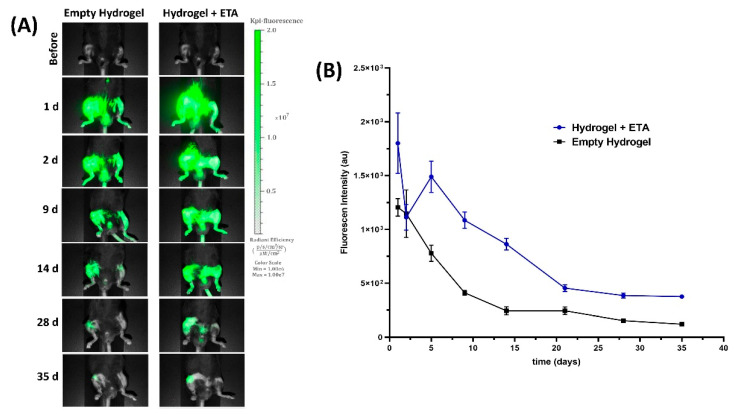
Mice knee images after hydrogels injection (**A**) NIR fluorescence imaging of mice knee joints taken at different time points after intra-articular injection of empty and ETA-loaded hydrogels containing NIR-780. (**B**) Graph depicting the decrease of fluorescence intensity after injection over the duration of the experiment. Data are presented as mean ± SD (*n* = 6).

Our intra-articularly injected hydrogels were labeled with IR-780 to observe their retention at the application site. Mice were scanned before they were injected with the hydrogels and also on day 2, 9, 14, 28, and 35 after being injected using the Pearl Impulse Imaging System at 800 nm (Figure 5A). Although a gradual decrease in the intensity of the fluorescent signal was observed in both hydrogels (empty and ETA-loaded), a distinct fluorescence signal was detectable in vivo over the course of 35 days, which shows that the materials remained inside the joint until the end of the study. In Figure 5B, the intensity of the fluorescence signal is plotted over the duration of the experiment. In the graph, it can be observed that the intensity of the fluorescence signal of ETA-loaded hydrogel was 5 times lower at the end of the study (day 35) than at the beginning (day 1). Regarding the empty hydrogel, the loss of fluorescence signal was even more pronounced, dropping by as much as 10 times at the end of the study (day 35) when compared to the beginning (day 2).

### 2.5. µ-CT and Histological Analysis

**Figure 6:**
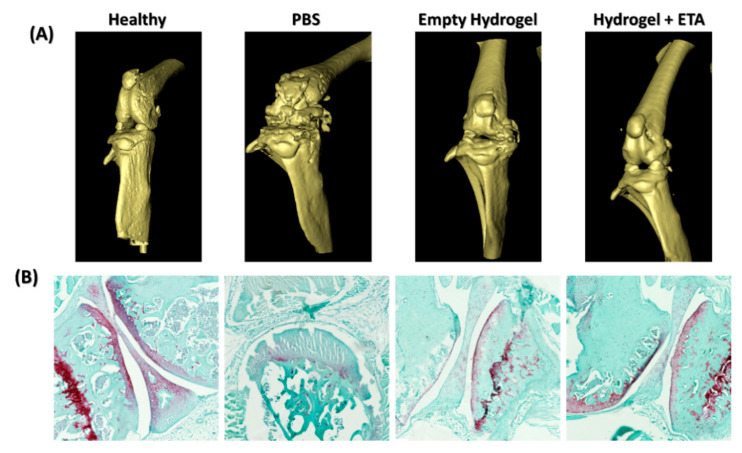
Representative mouse knee images in µ-CT and histological analysis (**A**) In vivo µ-CT scans of mouse knee joints showing a 3D view of the cartilage surface. From left to right healthy knee (negative control), OA knee with PBS injection (sham treatment/positive control), OA knee treated with empty hydrogel, and OA knee treated with ETA-loaded hydrogel. (**B**) Histological staining with Safranin O/Fast Green of sections from healthy, sham-treated, and hydrogel-treated mouse knees.

As is well known, OA is a progressive disease that occurs not only with damage to the articular cartilage but is also accompanied by osteochondral destruction [55]. To evaluate the effects of empty and ETA-loaded hydrogels on cartilage breakdown and bone erosion, µ-CT scans were performed. Figure 6A shows the image of a healthy mouse knee joint from the control group, which was not induced with OA. This serves as a reference for comparison with mice from the OA groups. As can be seen in the group injected with PBS (i.e., sham treatment), the cartilage and bone of the knee joints of these mice exhibited advanced degeneration. Importantly, hydrogel-treated mice did not show this kind of advanced cartilage and bone degeneration. In addition, it can be seen that the damage in knees injected with the ETA-loaded hydrogel was slightly more reduced compared to knees injected with the empty hydrogel. This shows ETA prevents the process of cartilage degeneration.

The therapeutic effect of the injected hydrogels was further evaluated by histological examination using Safranin O/Fast Green staining (Figure 6B), which allows for the visualization of normal cartilage (light red) and subchondral bone (light green) [56]. Safranin O staining was used to reveal changes that occurred in the articular cartilage, since the red color and intensity of the staining was proportional to the content of proteoglycans in the cartilage [57,58]. The healthy knee in Figure 6B possesses a smooth cartilage surface without lesions as well as strong Safranin O staining. In contrast, the PBS-treated OA knee shows a surface with prominent erosion areas along with decreased Safranin O staining. This demonstrates almost a total loss of proteoglycan content and suggests severe destruction accompanied by an extensive loss of cartilage. In the hydrogel-treated mice (empty and ETA-loaded), damage to the intra-articular cartilage was severely reduced. In both cases, the surface was smooth, not eroded, and Safranin O staining was prominent in the outermost tissue, demonstrating the presence of proteoglycans. However, the mice treated with the ETA-loaded hydrogels showed a more prominent Safranin O staining with intra-articular cartilage tissue that was consistently more uniform and similar to that of healthy mice.

The µ-CT scans as well as the ex vivo results demonstrate two things, namely (i) on its own, hydrogel has a regenerative effect on cartilage, which may be due to the presence of chitosan in our formulation and (ii) the addition of ETA inhibits degeneration and might even induce intra-articular cartilage regeneration. Furthermore, our conclusion is supported by previous in vitro and in vivo studies that demonstrated that chitosan promotes the expression of cartilage matrix compounds, reduces the production of inflammatory and catabolic mediators by chondrocytes [59,60], and intra-articular application prevents cartilage degradation and inflammation of the synovial membrane [61]. On the other hand, ETA is an inhibitor of the proinflammatory cytokine TNF-α, which plays an important role not only in inflammatory arthritis but also in degenerative joint disease [62,63]. The catabolic effect of TNF-α can induce bone resorption and cartilage destruction. Therefore, the inclusion of ETA in the hydrogel could reduce the levels of TNF-α in the joint, thereby halting, slowing down or preventing degenerative processes from being triggered and achieving beneficial results, such as those obtained in our present study.

## 3. Conclusions

In this study, we prepared two injectable, thermosensitive hydrogel formulations, characterized them, and were able to prove that they are cytocompatible with human chondrocytes and can control release of ETA in vitro over a period of 7 days. In particular, the ETA-loaded CS/PF/BGP hydrogel was applied for the treatment of knee joint OA in mice. In addition, it was determined that the maximum ETA concentration in blood was reached 24 h after injection of free ETA in PBS and 48 h after injection of the ETA-loaded hydrogel, thus demonstrating the ability of the material to delay the release in vivo as well. Additionally, the ex vivo investigation showed that the hydrogel itself had a protective effect on cartilage. Cartilage protection was enhanced by the incorporation of ETA into the material. Therefore, the results demonstrate that the intra-articular injection of ETA-loaded CS/PF/BGP hydrogel as a localized drug delivery system has great prospects in the treatment of knee OA.

## 4. Materials and Methods

Commercial chitosan from shrimp shells (low viscosity), Pluronic F127 (M.W. = 12,600), and sodium β-glycerolphosphate (glycerol 2-phosphate disodium salt hydrate) were provided by Sigma-Aldrich (San Luis, MO, USA). Etanercept (Embrel) was obtained from Pfizer BV (The Netherlands). Dulbecco’s modified eagles’ medium (DMEM, high glucose, with Glutamax^TM^), fetal bovine serum (FBS), penicillin, and streptomycin were purchased from Life Technologies (Breda, The Netherlands). 3-(4,5-Dimethylthiazol-2-yl)-5-(3-carboxymethoxyphenyl)-2-(4-sulfophe-nyl)-2H-tetrazolium (MTS, Promega, Madison, WI, USA) and a calcein-AM/ethidium homodimer-1 LIVE/DEAD^®^ assay kit (Invitrogen) were obtained from Carlsbad, CA, USA.

### 4.1. Hydrogel Preparation

The chitosan/Pluronic (CS/PF) hydrogels were prepared following the previously established methodology [29]. Briefly, a solution of 1.2% (*w*/*v*) CS in 1% (*v*/*v*) acetic acid was prepared, the pH was adjusted to approximately 6.4 with NaOH 0.1 mol/L and allowed to cool down. Then, PF was added for a final concentration of 20% (*w*/*v*). The mixture was constantly stirred at 4 °C overnight until complete dissolution. To achieve the cross-linked hydrogel, a given amount of BGP was dissolved in water and then added dropwise to the CS/PF solution under magnetic stirring at 4 °C. The final sample had a BGP concentration of 10% (*w*/*v*) (hydrogel CS/PF/BGP).

### 4.2. Hydrogel Characterization

#### 4.2.1. Gelation Time

The transition time from liquid to gel phase, namely the gelation time, was determined by the vial tilting method [41,64]. A total of 1 mL of hydrogel solution was placed in 15 mL tubes and incubated at 37 °C in a thermostatic bath for 1 min. The tube was removed every 30 s and inverted to record gel formation. The gelation time was established based on the criterion that the gel did not flow for at least 15 s after inverting the tube. Each sampling procedure was performed in triplicate. For comparison, PF hydrogel was used as a reference.

#### 4.2.2. Morphological Characterization

The morphology of the hydrogels was investigated by performing scanning electron microscopy (SEM). Freeze-dried hydrogels were sectioned, mounted on metal stubs, and sputter-coated with Pt/Pd (Cressington 208HR, Watford, UK). The samples were observed in a NanoSEM 200 electron microscope (FEI, Tokyo, Japan) at an acceleration voltage of 10 kV.

#### 4.2.3. Fourier Transform Infrared Spectroscopy (FTIR)

FTIR spectra of the raw materials and freeze-dried hydrogels were obtained using the Shimadzu IRSpirit-T FTIR spectrophotometer (Kyoto, Japan) in conjunction with the attenuated total reflectance (ATR), sampling accessory to allow for analysis without any sample preparation. The experiments were performed in the mid-infrared region from 4000 to 400 cm^−1^ at a resolution of 4 cm^−1^ accumulating 32 scans.

### 4.3. In Vitro Experiments

#### 4.3.1. ETA Release Study

To carry out the release study, 0.5 mL of each hydrogel was loaded with 25 µg of ETA in 5 mL vials and incubated at 37 °C for 10 min to ensure complete gelation. Next, 2 mL of PBS was added as release medium and the vial was placed back at 37 °C to perform the study. At specified times, a 0.2 mL sample of the supernatant was collected with help of p200 micropipette (10–200 µL), and an equal volume of fresh pre-warmed PBS at 37 °C was carefully added to the vial. The extracted sample was analyzed by performing UV-Vis spectrophotometry (NanoDrop 1000 One Microvolume, Thermo Scientific, Oslo, Norway) at 280 nm. The cumulative amount of released ETA was calculated with reference to a prepared standard calibration curve. Three independent release experiments were performed for each hydrogel composition.

From the data obtained, a study of the release behavior was conducted, applying mathematical models that describe possible mechanisms through which the drug was released from the matrix.

First, the Higuchi model [65] was applied, which is a time-dependent process and is based on Fick’s law of diffusion.

$$\frac{M_{t}}{M_{\infty }}=K\cdot t^{1/2}$$

where *M_t_/M_∞_* is the amount of drug released on time *t* and *K* is the release constant.

Next, the Korsmeyer–Peppas model [66,67] was applied, in which the *n* term was introduced; it is related to the release mechanism depending on the morphology and type of the studied material.

$$\frac{M_{t}}{M_{\infty }}=K\cdot t^{n}$$

where *M_t_/M_∞_* is the amount of drug released on time *t*, *K* is the release velocity constant, and *n* is the exponent of release as a function of time *t*.

Then, the Peppas–Sahlin model [68] was applied, which combines the release due to a Fickian diffusion and the substrate relaxation mechanisms, resulting in an abnormal release method.

$$\frac{M_{t}}{M_{\infty }}=K_{1}\cdot t^{m}+K_{2}\cdot t^{2m}$$

where *M_t_/M_∞_* is the fraction of drug released from the scaffold at time *t. K_1_* is the kinetic constant for the Fickian contribution of drug release. *K_2_* is the kinetic constant for the substrate relaxation, while *t* represents the release time, and *m* is the diffusional exponent.

#### 4.3.2. Hydrogel Cytocompatibility

The cytotoxicity of the prepared hydrogel systems was investigated in the presence of human chondrocyte cells (C28/I2 cell line) using the 3-(4,5-dimethylthiazol-2-yl)-5-(3-carboxymethoxyphenyl)-2-(4-sulfophenyl)-2H-tetrazolium (MTS) colorimetric method [69]. C28/I2 cells were grown in Dulbecco’s modified Eagle’s medium (DMEM) supplemented with 10% fetal bovine serum and 1% w/v antibiotics consisting of streptomycin (100 µg/mL) and penicillin (100 IU/mL). The assay was carried out using a procedure described elsewhere [29]. Briefly, 1 × 10^4^ cells were seeded onto the freeze-dried hydrogels placed in 96-well plates, and cultured in a standard cell culture incubator with 5% CO_2_ at 37 °C. After each prescribed incubation time (24, 48, and 72 h), 20 µL of MTS was added to each well. The plates were allowed to stand in the dark for 3 h, and then 100 µL of the supernatant was removed and transferred to a new 96-well plate. Cells without hydrogel served as a negative control and were considered to have 100% viability. Optical density (OD) was determined using a microplate reader (VersaMax equipped with Softmax Pro, Molecular Devices, San Jose, CA, USA) at a wavelength of 490 nm. Cell viability is expressed as the percentage of live cells with respect to the negative control. The cytotoxicity results were expressed as the percentage of viable cells according to the following equation:

$$\mathrm{Cell}\mathrm{viability}\left(\%\right)=\frac{{\mathrm{OD}}_{490\;\left(\mathrm{sample}\right)}}{{\mathrm{OD}}_{490\;\left(\mathrm{control}\right)}}\times 100$$

where *OD*_490(*sample*)_ is the absorbance obtained from the wells containing hydrogels and *OD*_490(*control*)_ is the absorbance obtained from the wells containing cells without hydrogels.

#### 4.3.3. LIVE/DEAD Staining on Hydrogels

To study the cell viability of C28/I2 chondrocytes at longer incubation times against the hydrogels, the live/dead assay was used. Cells were seeded on the hydrogels and incubated for 7 and 10 days in 48-well plates. Afterwards, the culture medium was removed, the hydrogels were washed three times with PBS, and the cells were incubated with an aliquot of the working solution for 45 min. The working solution was prepared according to the manufacturer’s instructions, adding 1 µL of calcein-AM stock solution and 4 µL of ethidium homodimer-1 (EthE-1) in 2 mL of PBS. At the end of the incubation, the working solution was removed, the hydrogels were washed with PBS, and the samples were observed using a confocal microscope (Leica TCS SP5, Leica Microsystems BV, Amsterdam, The Netherlands) with 450–490 nm excitation filters (green, calcein AM) and 510–560 nm (red, EthE-1). Live cells were seen in green and dead cells in red.

### 4.4. In Vico and Ex Vivo Experiments

#### 4.4.1. Animal Models

The animal studies were carried out at the animal facilities of the Leiden University Medical Center (LUMC) and were approved by the Animal Welfare Committee (permit: AVD1160020171405; PE.18.101.002), complying with the Dutch National Law on animal experiments. Twenty-nine 12-week-old male C57BL/6Jico mice were purchased from Charles River Laboratories (Charles River, Chatillon-sur-Chalaronne, France) for this study. Animal care and handling were performed in accordance with the guidelines and regulations as stipulated by the Dutch Experiments on Animals Act (WoD) and the European Directive on the Protection of Animals Used for Scientific Purposes (2010/63/EU). All applicable institutional and national guidelines for the care and use of animals were followed. Mice were kept under standard housing conditions at the Animal Facility of Leiden University Medical Center (LUMC, the Netherlands; group cages with enriched environment, food and water ad libitum; diurnal light cycle (12h light, 12h dark), temperature 21 °C; humidity 60%). The mice were divided into the following 5 groups: a negative control group of 5 mice that remained healthy and another 4 groups (*n* = 6 mice/group) underwent surgical destabilization of the (right) knee Medial Meniscus (DMM) to induce osteoarthritis (OA). Fourteen days post-surgery, operated mice were injected intra-articularly with (i) positive control (PBS), (ii) ETA (100 µg/mL), (iii) empty hydrogel, or (iv) hydrogel with ETA (100 µg/mL). All mice received an intra-articular injection of 6 µL according to the specific treatment of the group. Iodide dye IR-780 (Sigma-Aldrich, Amsterdam, the Netherlands) was added to the hydrogels at a concentration of 30 µg/mL to observe the retention properties of the materials in the joint.

#### 4.4.2. In Vivo ETA Release

To follow ETA release after intra-articular injection in vivo, blood was drawn via tail vein cuts at 1, 2, 3, 6, 9, and 12 days after injection. The blood was centrifuged at 7000× *g* for 5 min to obtain serum, and the serum ETA concentrations were determined by an enzyme-linked immunosorbent assay (ELISA) using a standard calibration curve.

#### 4.4.3. In Vivo Imaging

At different pre-established times, the intensity of the IR-780-iodine dye incorporated into the injected hydrogels was measured in order to investigate the retention of hydrogels in the mouse knee joint. Mice were scanned using the Pearl Impulse Imaging System (Li-Cor, Lincoln, NE, USA). The images of the mice knees were acquired and analyzed with the 800 nm emission filter. The study was carried out for 35 days, and for image acquisition the mice were anesthetized with isoflurane balanced with oxygen.

The mice were scanned using a Skyscan 1076 Micro-CT scanner (Skyscan, Kontich, Belgium) at a voltage of 50 kV and current of 200 µA, with an X-ray source rotation step size of 1° and 180°. Scans were performed under injection anesthesia (ketamine 100 mg/kg and xylazine 12.5 mg/kg). Images were taken with an image pixel resolution of 9 µm and an average frame of three to reduce noise. Reconstructions were performed using nRecon V1.6.2.0 software (Skyscan) with beam hardening correction set to 10%, ring artifact correction set to 10, and dynamic range set to −1000–4000 Hounsfield units.

#### 4.4.4. Histological ANALYSIS

After isolation, the mice knee joints were fixed with buffered paraformaldehyde, decalcified for two weeks and embedded in paraffin. Subsequently, they were cut into 10 µm sections and stained with Safranin O/Fast Green [58] [, to evaluate the cartilage damage. Slides were observed using light microscopy with an Axio Scan.Z1 (Zeiss, Jena, Germany) equipped with a Colibri LED light source, Hitachi BF color camera and an ORCA-Flash sensitive camera for fluorescence imaging. The collected images were processed with Zen 3.3 software (blue edition).

### 4.5. Statistical Analysis

Graphs and statistics were obtained with OriginPro 2021 (OriginLab Corp., Northampton, MA, USA). Data are reported as mean ± standard deviation (SD), unless stated otherwise. Error bars represent the SD calculated from tests of triplicate measurements for each sample. The differences between the groups were examined with one-way and two-way analysis of variance (ANOVA) for *p* < 0.05 or *p* < 0.01, according to the t-test for two samples or a multiple samples’ comparison.

## Figures and Tables

**Table 1:** Summary of the fit parameters to the release models for the hydrogels.

Samples		CS/PF	CS/PF/BGP
Model	Parameters		
Higuchi	*K*	31 ± 1	19 ± 2
	*R* ^2^	0.9555	0.7655
Korsmeyer-Peppas	*K*	35 ± 2	23 ± 2
	*n*	0.41 ± 0.03	0.33 ± 0.06
	*R* ^2^	0.9762	0.8531
Peppas-Sahlin	*K*_1_	44 ± 3	33 ± 4
	*K*_2_	−7 ± 1	−7 ± 2
	*n*	0.56 ± 0.04	0.5 ± 0.1
	*R* ^2^	0.9864	0.8902

## Data Availability

The data presented in this study are available on request from the corresponding authors.
